# Supplementary material for: The control of transcriptional memory by stable mitotic bookmarking
Source: Nat Commun. 2022 Mar 4;13:1176. doi: 10.1038/s41467-022-28855-y (PMC8897465; doi:10.1038/s41467-022-28855-y)
Supplement: Supplementary file 3 — Description of Additional Supplementary Files [file 41467_2022_28855_MOESM3_ESM.pdf]

Description of Additional Supplementary Files

File Name: Supplementary Data 1
Description: **Primers sequences for cloning, DNA-FISH and smFISH probes sequences used in this study.**

File Name: Supplementary Data 2
Description: **Identified GAF mitotically retained, interphase only and mitotic only peak coordinates, the nearest gene and the distance to the respective nearest TSS.**

File Name: Supplementary Data 3
Description: **Identified GAF mitotically retained peaks coordinates and the respective features for each peak.**

File Name: Supplementary Data 4
Description: **Results of the fit of the homogeneous jump model to the live imaging data performed in this study.**

File Name: Supplementary Movie 1
Description: **Imaging GAF behavior during the cell cycle**. Maximum intensity projection of confocal live imaging of a developing His2Av-mRFP;GAF-GFP embryo. Movies comprise 15 Z-planes projected images, representing whole nuclei. Left panel is green channel only (GAF-GFP), middle panel is the red channel only His2Av-mRFP and merge channel is on the right panel. Scale bar is 5µm.

File Name: Supplementary Movie 2
Description: **GAF subnuclear localization.** Maximum intensity projection of confocal live imaging of His2Av-mRFP;GAF-GFP embryo. Top movie comprises a six Z-planes projected images at the apical side of nuclei, bottom movie is a six Z-planes projected images at the basal side of nuclei. This movie shows the enrichment of GAF big nuclear puncta on the apical side of the nuclei. Heatmap color scales to the intensity of GAF-GFP signal. Time is in minutes. Scale bar is 5µm.

File Name: Supplementary Movie 3
Description: **Transcription of scylla in a wild-type embryo (ventral view).** Maximum intensity projection of confocal live imaging of a MCP-eGFP, His2Av-mRFP/+ > scylla_24XMS2_CRISPR/+ embryo. Nuclei are visualized in red and scylla transcription in green, from nuclear cycle 12 until the onset of gastrulation. Movie is taken in the middle of the ventral side of the embryo (as shown in Figure 4b). Scale bar is 5µm.

File Name: Supplementary Movie 4
Description: **Transcription of scylla in a wild-type embryo (dorsal view).** Maximum intensity projection of confocal live imaging of a MCP-eGFP, His2Av-mRFP/+ > scylla_24XMS2_CRISPR/+ embryo. Nuclei are visualized in red and scylla transcription in green, from nuclear cycle 13 until the appearance of the germ band extension. Movie is taken in the middle of the dorsal side of the embryo (as shown in Supplementary Figure 6c). Scale bar is 5µm.

File Name: Supplementary Movie 5
Description: **Transcription of scylla in a control embryo.** Maximum intensity projection of confocal live imaging of a mat-alphaTub-Gal4/+; nos-Gal4, MCP-eGFP, His2Av-mRFP/UASp-shRNAwhite > scylla_24X-MS2_CRISPR/+ embryo. To maximize RNAi efficiency, we used two maternal GAL4 drivers, namely

materna-alpha-Tubulin-Gal4 (mat-alphaTub-Gal4) and nanos-Gal4 (nos-Gal4). Nuclei are visualized in red and scylla transcription in green, from nuclear cycle 13 (nc13) to nuclear cycle 14 (nc14) and until gastrulation furrow appears. Scale bar is 10µm.

File Name: Supplementary Movie 6
Description: **Transcription of scylla in a GAF maternally depleted embryo**. Maximum intensity projection of confocal live imaging of a mat-alphaTub-Gal4/+; nos-Gal4, MCP-eGFP, His2AvmRFP/UASp-shRNA-GAF > scylla_24X-MS2_CRISPR/+ embryo. Nuclei are visualized in red and scylla transcription in green, from nuclear cycle 13 (nc13) to nuclear cycle 14 (nc14). Maternal GAF is depleted using maternal-alphaTubulin-Gal4 driver (see methods) to express shRNA-GAF in the ovary. As in GAF depleted embryos gastrulation in not always complete, we imaged until at least 1h after the mitosis. Scale bar is 10µm.
