# Supplementary material for: The control of transcriptional memory by stable mitotic bookmarking
Source: Nat Commun. 2022 Mar 4;13:1176. doi: 10.1038/s41467-022-28855-y (PMC8897465; doi:10.1038/s41467-022-28855-y)
Supplement: Supplementary file 14 — Reporting Summary [file 41467_2022_28855_MOESM14_ESM.pdf]

nature portfolio

Corresponding author(s): Mounia Lagha
Last updated by author(s): 02/03/2022

Reporting Summary

Nature Portfolio wishes to improve the reproducibility of the work that we publish. This form provides structure for consistency and transparency in reporting. For further information on Nature Portfolio policies, see our Editorial Policies and the Editorial Policy Checklist.

Statistics

For all statistical analyses, confirm that the following items are present in the figure legend, table legend, main text, or Methods section.

n/a	Confirmed	
☐	☒	The exact sample size (*n*) for each experimental group/condition, given as a discrete number and unit of measurement
☐	☒	A statement on whether measurements were taken from distinct samples or whether the same sample was measured repeatedly
☐	☒	The statistical test(s) used AND whether they are one- or two-sided<br>*Only common tests should be described solely by name; describe more complex techniques in the Methods section.*
☒	☐	A description of all covariates tested
☒	☐	A description of any assumptions or corrections, such as tests of normality and adjustment for multiple comparisons
☐	☒	A full description of the statistical parameters including central tendency (e.g. means) or other basic estimates (e.g. regression coefficient) AND variation (e.g. standard deviation) or associated estimates of uncertainty (e.g. confidence intervals)
☐	☒	For null hypothesis testing, the test statistic (e.g. F, t, r) with confidence intervals, effect sizes, degrees of freedom and P value noted<br>*Give P values as exact values whenever suitable.*
☒	☐	For Bayesian analysis, information on the choice of priors and Markov chain Monte Carlo settings
☒	☐	For hierarchical and complex designs, identification of the appropriate level for tests and full reporting of outcomes
☒	☐	Estimates of effect sizes (e.g. Cohen's *d*, Pearson's *r*), indicating how they were calculated

Our web collection on statistics for biologists contains articles on many of the points above.

Software and code

Policy information about availability of computer code

Data collection	Confocal microscopes with ZEISS Zen black software: LSM780: ZEN 2012 SP1 Black Edition - Version 8.1.3.484, LSM880: ZEN 2.3 SP1 FP1 Black - Version 14.0.9.201
Data analysis	GraphPad Prism 8.0.1 was used for figures and statistical tests.<br>Reads from ChIP-seq and Input experiments were trimmed for quality using Cutadapt (v1.16).<br>To measure the distances between probes (scyl-chrb and chrb-ctrl, or esg-sna and sna-ctrl), we used<br>a custom-made software developed in PythonTM programming language version 2.7.15rc1 (with relative packages). This software is available through this link: https://github.com/ant-trullo/DNA_FishAnalyzer.<br>For Fluorescence Correlation Spectroscopy we used PyCorrFit (https://pycorrfit.readthedocs.io/en/stable/sec_about.html).<br>For FRAP curve fitting we used MatLab 2014b (Mathworks Inc. USA).

For manuscripts utilizing custom algorithms or software that are central to the research but not yet described in published literature, software must be made available to editors and reviewers. We strongly encourage code deposition in a community repository (e.g. GitHub). See the Nature Portfolio guidelines for submitting code & software for further information.

Data

Policy information about availability of data

All manuscripts must include a data availability statement. This statement should provide the following information, where applicable:

- Accession codes, unique identifiers, or web links for publicly available datasets
- A description of any restrictions on data availability
- For clinical datasets or third party data, please ensure that the statement adheres to our policy

All relevant data supporting the key findings of this study are available within the article and its Supplementary Information files or from the corresponding author upon reasonable request. All ChIP-seq data and bed files are accessible at GEO Accession viewer under the number: GSE180812. The updated version of MitoTrack is available at: https://github.com/ant-trullo/MitoTrack_v4_0. The distance measurement software is available through this link: https://github.com/ant-trullo/DNA_FishAnalyzer.

Field-specific reporting

Please select the one below that is the best fit for your research. If you are not sure, read the appropriate sections before making your selection.

- [x] Life sciences
- [ ] Behavioural & social sciences
- [ ] Ecological, evolutionary & environmental sciences

For a reference copy of the document with all sections, see nature.com/documents/nr-reporting-summary-flat.pdf

Life sciences study design

All studies must disclose on these points even when the disclosure is negative.

Sample size	Sample sizes were determined accordingly to typical sample size in the field (Dufourt et al. 2018, Pimmett et al. 2021). Experiments were replicated to ensure statistical validity.
Data exclusions	For FRAP, data were excluded when they could not be accurately fitted by the analytical model (based on RMSE and chi2 test). For FCS, correlograms were discarded when the fluorescence trace (intensity fluctuation over time) was subjected to photobleaching. Photobleaching was monitored by changes in the average fluorescence value at long time compared to the average fluorescence value at the initial time of the recording. We used the same exclusion criteria as in Dufourt et al. 2018.
Replication	Each experiment was perfomed in 2-6 independent biological replicates. All attempts at replication were successful.
Randomization	Randomization is not necessary or applicable to our study as all of our data were well-controlled wild type vs mutant comparisons. Embryos were allocated based on genotypes.
Blinding	No blinding was performed in this study since our experiments did not require this (experiments are ran regarding genotypes).

Reporting for specific materials, systems and methods

We require information from authors about some types of materials, experimental systems and methods used in many studies. Here, indicate whether each material, system or method listed is relevant to your study. If you are not sure if a list item applies to your research, read the appropriate section before selecting a response.

Materials & experimental systems

n/a	Involved in the study	
[ ]	[x]	Antibodies
[x]	[ ]	Eukaryotic cell lines
[x]	[ ]	Palaeontology and archaeology
[ ]	[x]	Animals and other organisms
[x]	[ ]	Human research participants
[x]	[ ]	Clinical data
[x]	[ ]	Dual use research of concern

Methods

n/a	Involved in the study	
[ ]	[x]	ChIP-seq
[x]	[ ]	Flow cytometry
[x]	[ ]	MRI-based neuroimaging

Antibodies

Antibodies used	anti-GAF (gift from Dr. G.Cavalli, Schuettengruber et al. 2009)<br>anti-rabbit Alexa 488-conjugated (Life Technologies, A21206)<br>anti-mouse Alexa 488-conjugated (Life Technologies, A21202)<br>anti-rabbit Alexa 555-conjugated (Life Technologies, A31572)

	anti-sheep Alexa 555-conjugated (Life Technologies, A21436)<br>H3 (Ser10) antibody (Cell Signalling #9701)<br>anti-H4K8ac, abcam 15823<br>Anti-mouse and -rabbit IgG-HRP (Cell Signaling #7076 and #7074)<br>Anti-tubulin (Invitrogen, GT114)
Validation	All antibodies were validated by the manufacturers indicated above. The validation of these antibodies can found in the respective vendor websites.

Animals and other organisms

Policy information about studies involving animals; ARRIVE guidelines recommended for reporting animal research

Laboratory animals	All reported experiments were performed on embryos from transgenic and/or mutant variants of the Fruit Fly Drosophila melanogaster.
Wild animals	There were no wild animals used in this study.
Field-collected samples	There were no field-collected sample in this study.
Ethics oversight	No ethical approval was required

Note that full information on the approval of the study protocol must also be provided in the manuscript.

ChIP-seq

Data deposition

- [x] Confirm that both raw and final processed data have been deposited in a public database such as GEO.
- [x] Confirm that you have deposited or provided access to graph files (e.g. BED files) for the called peaks.

Data access links<br>*May remain private before publication.*	GSE180812.
Files in database submission	GSM5471314 ChIP_GAF_interphase_1<br>GSM5471315 ChIP_GAF_interphase_2<br>GSM5471316 CHIP_GAF_interphase_3<br>GSM5471317 ChIP_GAF_mitosis_1<br>GSM5471318 ChIP_GAF_mitosis_2<br>GSM5471319 input_GAF_interphase_1<br>GSM5471320 input_GAF_interphase_2<br>GSM5471321 input_GAF_interphase_3<br>GSM5471322 input_GAF_mitosis_1<br>GSM5471323 input_GAF_mitosis_2<br>GSM5471324 ChIP_H4K8ac_interphase_1<br>GSM5471325 ChIP_H4K8ac_interphase_2<br>GSM5471326 ChIP_H4K8ac_mitosis_1<br>GSM5471327 ChIP_H4K8ac_mitosis_2<br>GSM5471328 input_H4K8ac_interphase_1<br>GSM5471329 input_H4K8ac_interphase_2<br>GSM5471330 input_H4K8ac_mitosis_1<br>GSM5471331 input_H4K8ac_mitosis_2
Genome browser session<br>(e.g. UCSC)	No browser session has been created but all the data and the bed files are available under the accession number GSE180812.

Methodology

Replicates	Each conditions was done at least in duplicates.
Sequencing depth	Reads were pair-end (75bp). Total number of reads range from 10 330 357 to 20 473 302. Number of pair reads after quality trimming was between 10 211 499 to 20 298 184. Overall alignment rate was between 58,8% to 94%. Any extra information are available upon request to mounia.lagha@igmm.cnrs.fr
Antibodies	anti-GAF (gift from Dr. G.Cavalli, Schuettengruber et al. 2009)<br>anti-H4K8ac, abcam 15823
Peak calling parameters	Peak calling was done with a threshold of 100 for GAF-ChIP-seq and 22 for H4K8ac-ChIP-seq, minimum run of 50 bp and maximum gap of 200bp.
Data quality	We estimated a good correlation between replicates, therefore to call the enriched peaks from the final wiggle files, we used

	Thresholding function of the Integrated Genome Browser (IGB) to define the signal value over which we consider a genomic region to be enriched compared to background noise (Threshold).
Software	Both reads from ChIP-seq and Input experiments were trimmed for quality using a threshold of 20 and filtered for adapters using Cutadapt (v1.16). Reads shorter than 30 bp after trimming were removed. Reads were mapped to Drosophila melanogaster genome (dm6 release) using Bowtie2 (Langmead and Salzberg 2012). Aligned sequences were processed with the R package PASHA to generate the used wiggle files. Pasha elongates in sillico the aligned reads using the DNA fragment size estimated from paired-reads. Then, the resulting elongated reads were used to calculate the coverage score at each nucleotide in the genome. Wiggle files representing average enrichment score every 50 bp were generated. In order to normalize the enrichment scores to reads per million, we rescaled the wiggle files using PASHA package. Besides, in order to reduce the over-enrichment of some genomic regions due to biased sonication and DNA sequencing, we subtracted from ChIP sample wiggle files the signal present in Input sample wiggle files. The Rescaled and Input subtracted wiggle files from biological replicate were then used to generate the final wiggle file representing the mean signal.<br>In order to call the enriched peaks from the final wiggle files, we used Thresholding function of the Integrated Genome Browser (IGB) to define the signal value over which we consider a genomic region to be enriched compared to background noise (Threshold). We used also the minimum number of consecutive enriched bins to be considered an enriched region (Min.Run) as well as the minimum gap above which two enriched regions were considered to be distinct (Max.Gap). The three parameters were then used with an in-house script that realizes peak calling by using algorithm employed by Thresholding function of IGB.<br>Peaks calling was done with a threshold of 100 for GAF-ChIP-seq and 22 for H4K8ac-ChIP-seq, minimum run of 50 bp and maximum gap of 200bp. Interphase only peaks correspond to peaks from interphase ChIP-seq with no overlap with peaks from mitotic ChIP-seq. Mitotically retained correspond to interphase peaks with an overlap (min 1 base pair) with peaks from mitotic ChIP-seq. Mitotic only peaks correspond to peaks from mitotic ChIP-seq with no overlap with peaks from interphase ChIPseq.
