# Supplementary material for: The control of transcriptional memory by stable mitotic bookmarking
Source: Nat Commun. 2022 Mar 4;13:1176. doi: 10.1038/s41467-022-28855-y (PMC8897465; doi:10.1038/s41467-022-28855-y)
Supplement: Supplementary file 1 — Supplementary Information [file 41467_2022_28855_MOESM1_ESM.pdf]

Supplementary information

Supplementary Table 1: Whole-genome data used in this study

Experiment	Stage	Reference
TSS	-	GCF_000001215.4 (Release_6)
GAF-ChIP-seq	2-4 hr AEL, WT embryos	*Koenecke et al. 2017*
H3K27ac-ChIP-seq	2-4 hr AEL, tol10b embryos	*Koenecke et al. 2016*
H3K27ac-ChIP-seq	2-4 hr AEL, gd7 embryos	*Koenecke et al. 2016*
Enhancer reporter transgenes	Embryos	*Kvon et al. 2014*
Hi-C	nc12, nc13, nc14	*Hug et al. 2017*
Insulators	Embryos	*Negre et al. 2010*
ATAC-seq	nc8, 9, 10…13 every 3min	*Blythe & Wieschaus 2016*
ATAC-seq degrad-FP	2–2.5 hr AEL	*Gaskill et al. 2021*
H3K27ac	nc14	*Li et al. 2014*
H3K27me3	nc14	*Li et al. 2014*
RNA-seq	nc14	*Lott et al. 2011*

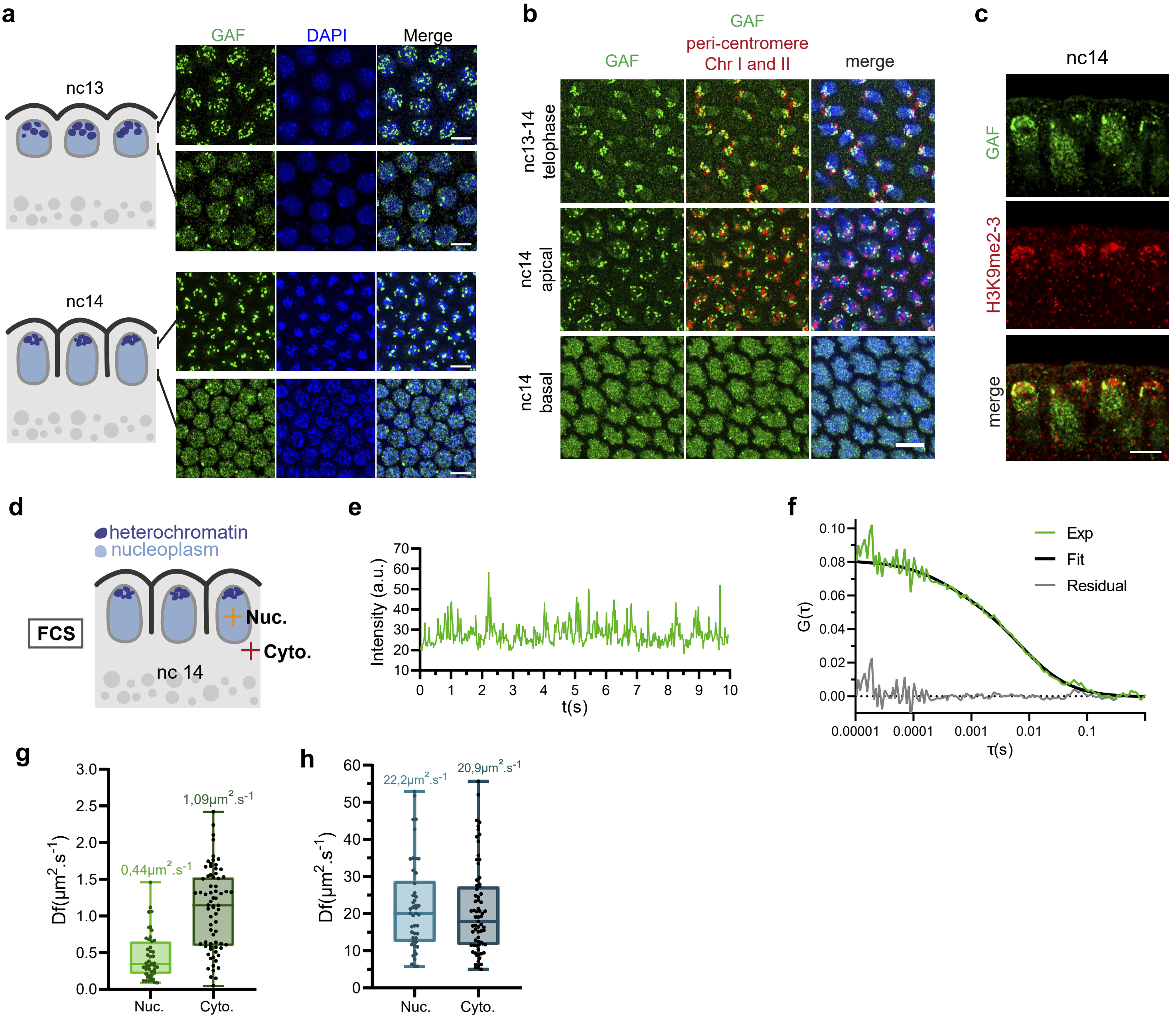

Supplementary Fig. 1. **GAF nuclear localization and kinetic properties. a** Maximum intensity projected Z-planes of confocal images showing GAF immunostaining (green) in wild-type embryos at the indicated stages counterstained with DAPI (blue). Upper panels are images taken at the apical side of the nuclei, bottom panels at the basal side. Scale bars are 5µm. **b** Maximum intensity projected Z-planes of confocal images from DNA-immunoFISH with peri-centromeric probes 80 (red) and anti-GAF (green) on wild-type embryos and at the indicated nuclear cycles. Scale bar is 5µm. **c** Maximum intensity projected Z-stack images from immunofluorescence of H3K9me2-3 (red) and GAF (green) on late n.c.14 wild-type embryos. Scale bar is 5µm. **d** Schematic of sagittal view of nc14 embryos. Nuclei are represented in light blue and apical hetero-chromatin regions in dark blue. Crosses represent regions targeted by FCS in the nucleoplasm (nuc.) and cytoplasm (cyto.), performed on GAF-GFP embryos. **e** Example of an intensity time trace obtained from FCS in a GAF-GFP nc14 embryo. **f** Example of autocorrelation function (green curve) related to (c) (black curve represents fitting using a double-diffusion model). **g** Estimated fast diffusion time for GAF-GFP extracted after fitting FCS data with a double-diffusion model performed in the nucleoplasm (nuc.) or in the cytoplasm (cyto.). 44 measures were taken in the nucleoplasm and 69 in the cytoplasm. Centered horizontal line of the box plot represents the median, whiskers represent min and max values. **h** Estimated slow diffusion time for GAF-GFP extracted after fitting FCS data with a double-diffusion model performed in the nucleoplasm (nuc.) or in the cytoplasm (cyto.). 44 measures were taken in the nucleoplasm and 69 in the cytoplasm. Centered horizontal line of the box plot represents the median, whiskers represent min and max values.

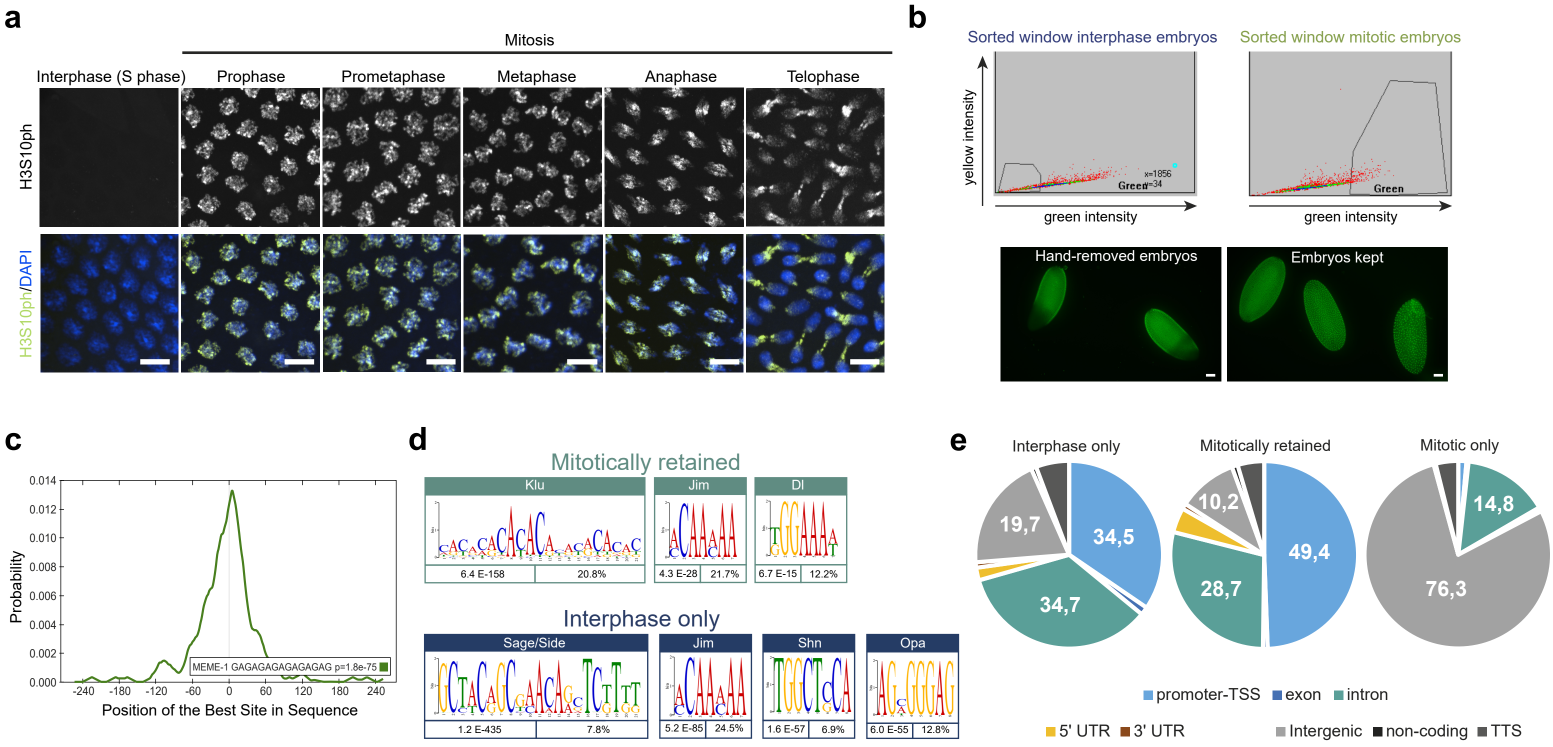

Supplementary Fig. 2. **Characterization of GAF mitotically retained regions. a** Maximum intensity projected Z-planes of confocal images of H3S10ph immunostaining (grey and green) in wild-type embryos at the indicated mitotic stages counterstained with DAPI (blue). Scale bars are 5µm. **b** (Top panels) Isolation of interphase and mitotic embryos previously stained with a H3S10ph antibody with a flow cytometer. Highlighted polygones show the range for selected embryos for the interphase population (blue) and the mitotic population (green). (Bottom panels) Representative embryos stained with an H3S10ph antibody, undergoing mitotic waves (left image) (removed during the hand sorting step) and fully mitotic embryos (right image). Scale bars are 50µm. **c** Profile of probability for the GAGAG motif reported by MEME within sequences from GAF-ChIP-seq peaks. **d** De-novo motif enrichment within GAF mitotically retained and interphase only peaks, as reported by MEME. **e** Pie charts representing the proportions of different regions annotated with HOMER tool in interphase only, mitotically retained and mitosis only GAF-ChIP-seq peaks.

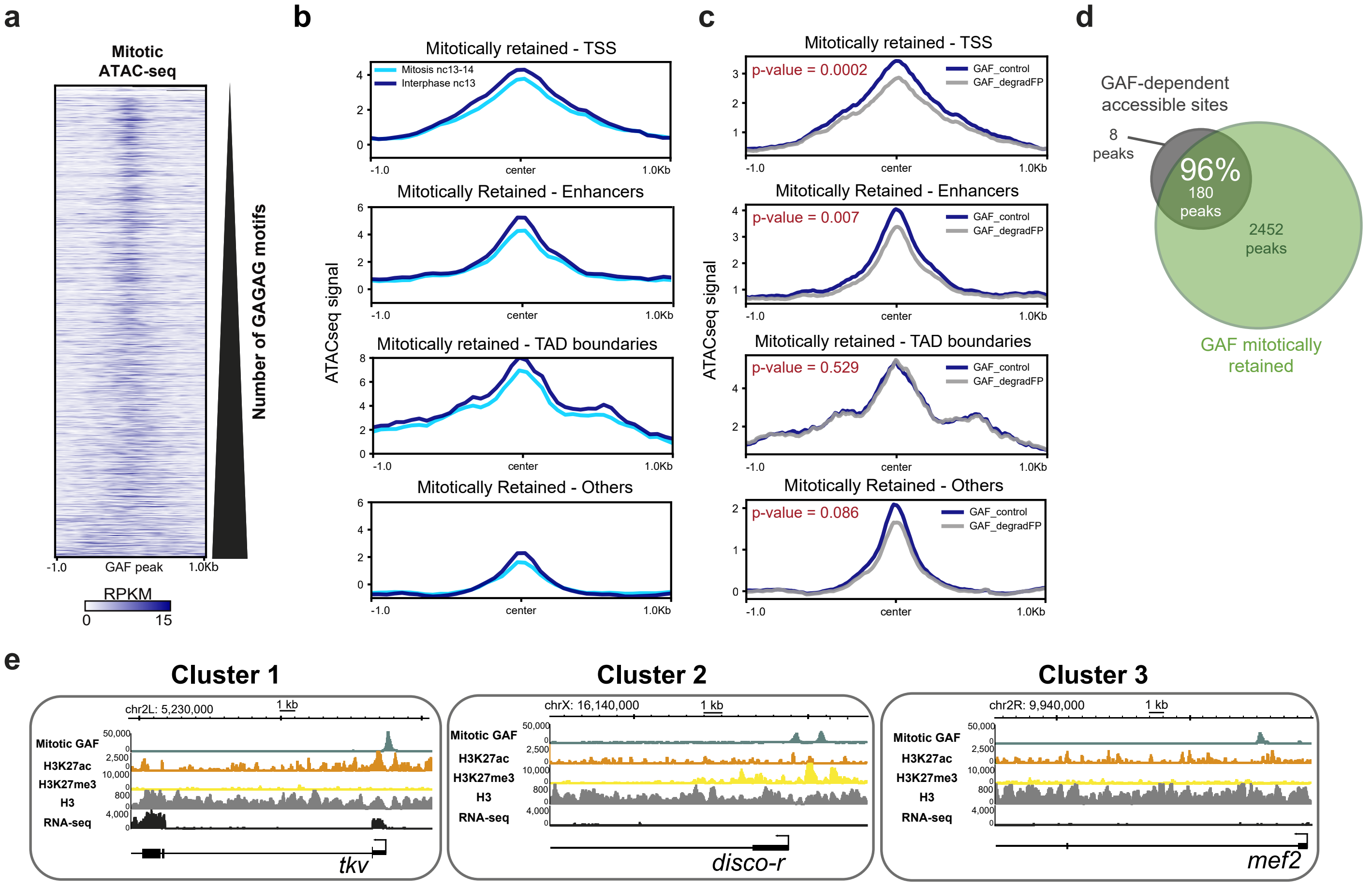

Supplementary Fig. 3. **Chromatin states of GAF mitotically retained regions. a** Heatmap of ATAC-seq signal 54 of interphase n.c.13 embryos at GAF mitotically retained regions, partitioned by the number of GAGAG motifs they contain. **b** Metagene profiles of the ATAC-seq signal 54 of interphase n.c.13 embryos at GAF mitotically retained regions partitioned by the type of cis-regulatory element they overlap. Plots show peaks located at TSS, TAD boundaries 55, enhancers 53,57 and others that were unassigned. **c** Metagene profiles of the ATAC-seq signal in control (GAF control, blue) or GAF depleted (GAF_degradFP, grey) 28 2-2.5h after egg laying embryos in different categories of GAF mitotically retained regions defined in Fig. 2b. P-values are from a two-sided Wilcoxon rank sum test with continuity correction, used to compare the curves of ATAC-seq accessibility in GAF_control and GAF_degradFP from-500bp to +500bp from the center of the peaks. **d** Venn diagram of the overlap between GAF dependent accessible loci 28 (grey) and GAF mitotically retained peaks (green). **e** Genome browser images showing ChIP-seq signals of mitotic GAF, H3K27ac, H3K27me3 and H3 57, alongside RNA-seq 58 at three loci containing GAF mitotically retained peaks. These three examples represent each of the three clusters defined by distinct chromatin states.

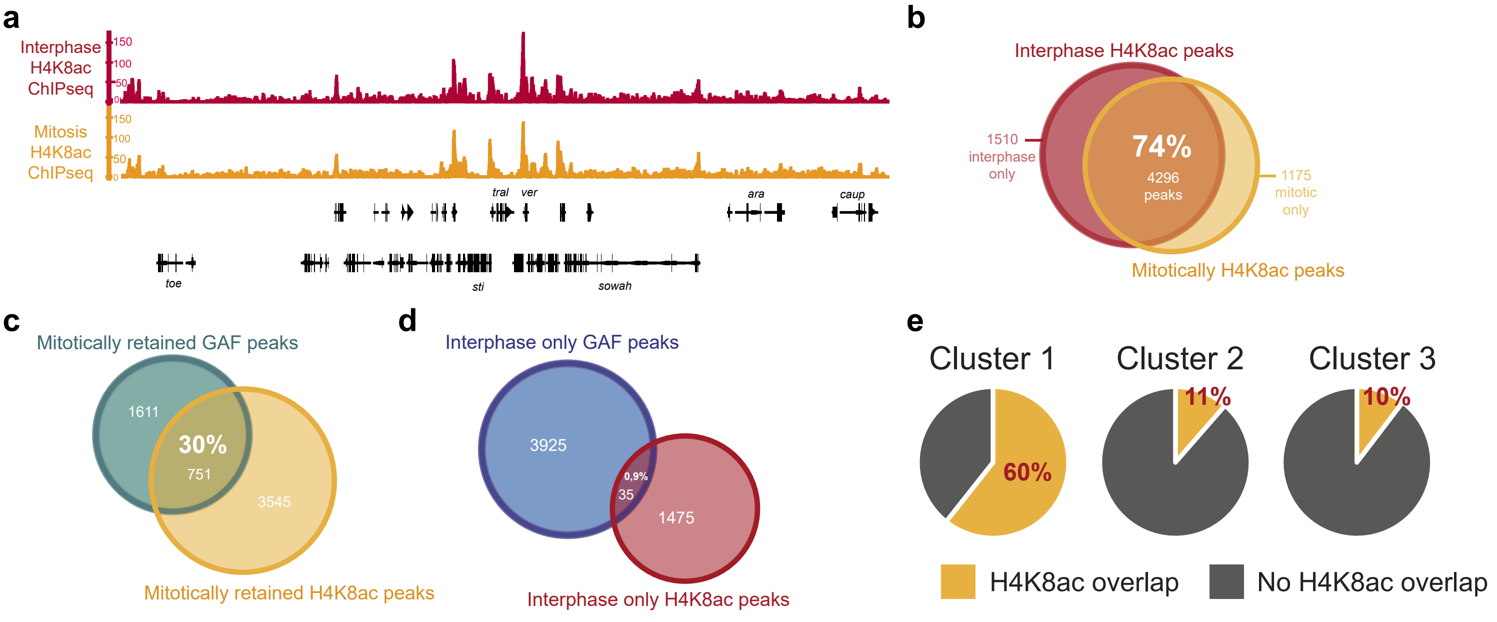

Supplementary Fig. 4. **H4K8ac-ChIP-seq in interphase and mitotic embryos. a** Genome browser image showing H4K8ac-ChIP-seq profiles of interphase and mitotic embryos at a representative genomic region containing mitotically retained H4K8ac peaks. **b** Venn diagram of the overlap between H4K8ac interphase (red) and mitotic (orange) ChIP-seq peaks. **c-d** Venn diagram of the overlap between mitotically retained (**c**) and interphase only (**d**) or H4K8ac and GAF-ChIP-seq peaks. **e** Pie charts of the overlap between H4K8ac-ChIP-seq mitotic peaks and GAF mitotically retained peaks, after their partitioning into three clusters.

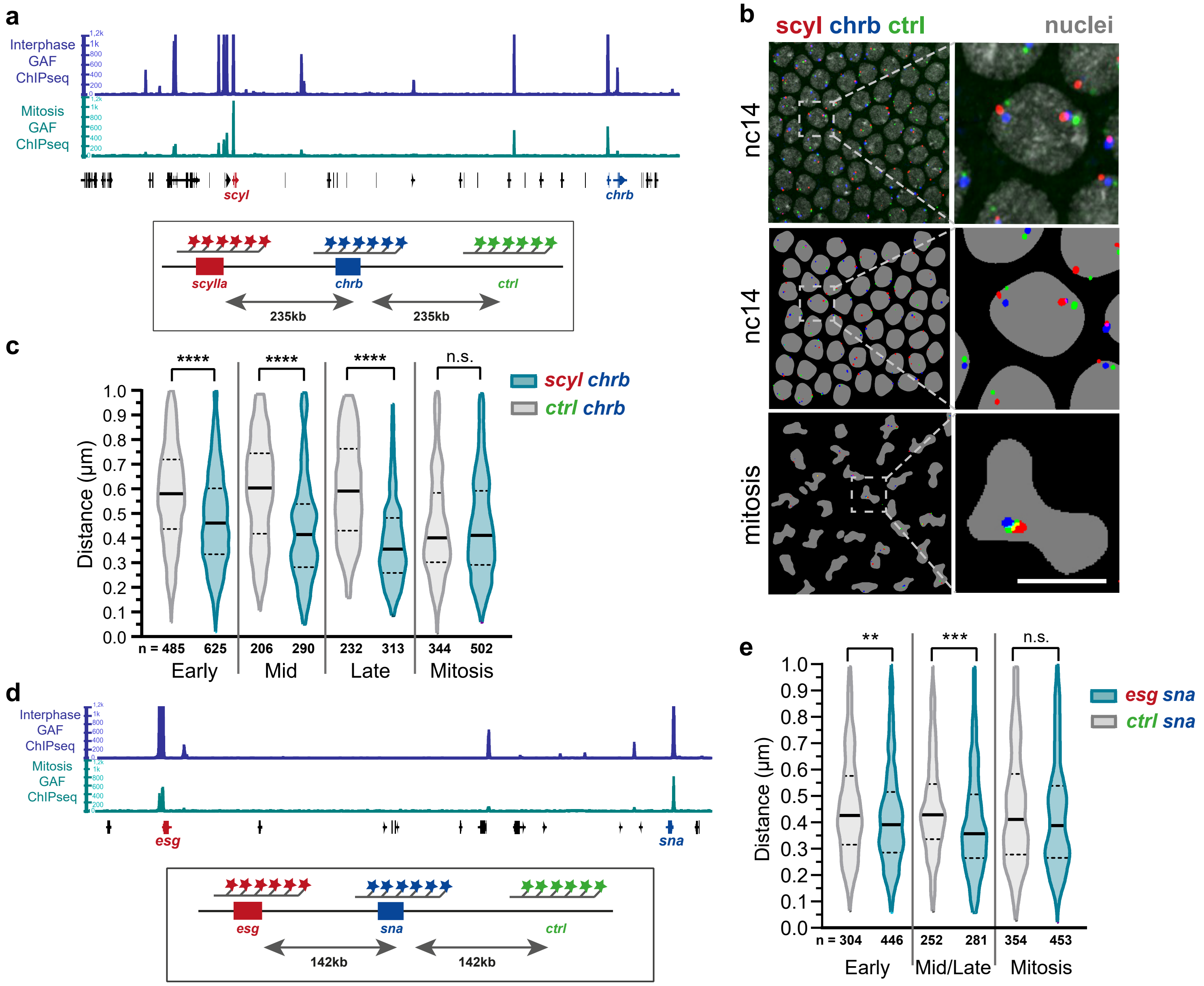

Supplementary Fig. 5. **Absence of mitotic loops for two GAF mitotically retained loci. a** (Top) Genome browser image of the interphase and mitotic GAF ChIP-seq profiles at the scyl-chrb locus. (Bottom) Schematic of the scyl-chrb locus with the indicated designed probes for DNA-FISH. The spatial distance between scyl and chrb was compared to that of a non-bookmarked region located at an equivalent distance (235kb). **b** DNA-FISH image in nc14 wild-type embryo with scyl labeled in red, chrb in blue and control region in green. Bottom images represent the same image false colored after analysis as well as nuclei in mitosis. Scale bar is 5µm. **c** Violin plot representing the distance between scyl-chrb and chrb-ctrl from images taken in early, middle, late n.c.14 interphase or mitotic wild-type embryos. Two tailed Welch's t-test ****p<0.0001. n.s. (non significative) p-value=0.8143. **d** (Top) Genome browser image of the interphase and mitotic GAF-ChIP-seq profiles at the sna-esg locus. (Bottom) schematic of the sna-esg locus with the positions of designed probes for DNA-FISH indicated. **e** Violin plot representing the distance between sna-esg and sna-ctrl from images taken in nc14 interphase or mitotic wild-type embryos. Two tailed Welch's t-test ***p=0.0003, **p=0.0043, n.s. (non significative) p=0.1076.

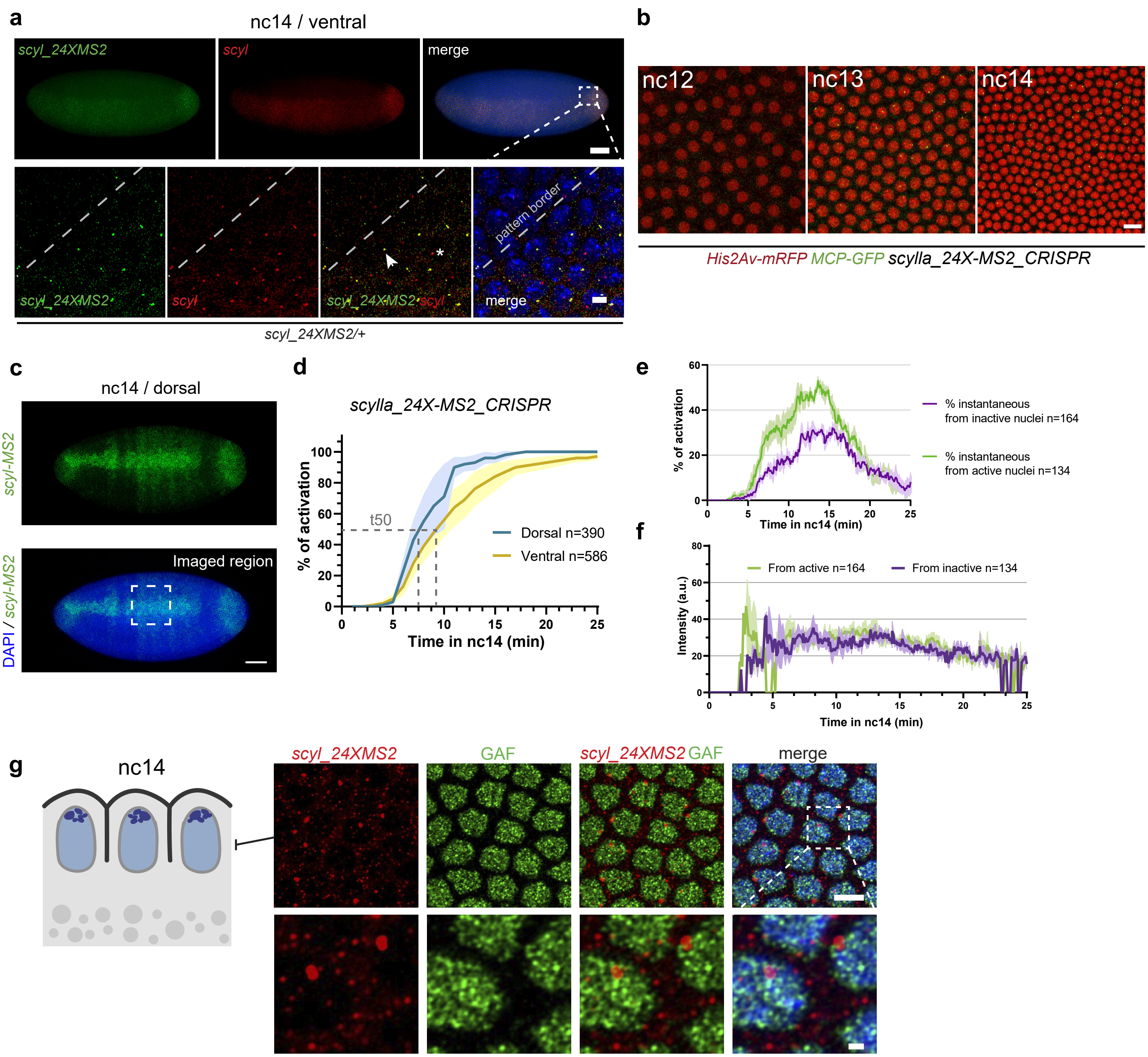

Supplementary Fig. 6. **Monitoring scylla transcription dynamics in the early embryo. a** Maximum intensity projected Z-planes of confocal images from smiFISH with MS2 probes (green) and scyl probes (red) counterstained with DAPI (blue) on wild-type embryos in early nc14 on the ventral side. scyl expression pattern is represented with a dashed grey line and shows that both scyl and MS2 expression follow the same pattern. Scale bar is 30µm on whole embryo image, and 5µm on the zoomed image. Arrowhead points scyl_24XMS2 locus recognized by both MS2 and scyl probes and the star a wild type locus (without 24XMS2). **b** Snapshots from a representative movie of scylla_24X-MS2_CRISPR/+ embryo carrying MCP-eGFP, His2Av-mRFP. Nuclei are visualized in red and transcription sites in green. Scale bar corresponds to 10µm (see related Supplementary Movie 3). **c** Maximum intensity projected Z-planes of confocal images from smiFISH with scyl probes (green) counterstained with DAPI (blue) on wild-type embryos in nc14 on the dorsal side. Dashed square represents the regions imaged for the quantifications shown in (d). Scale bars are 50µm. **d** Quantification of the transcriptional synchrony after mitosis in scylla_24X-MS2_CRISPR embryos in dorsal (blue) and ventral (yellow) regions. Both daughters of each nucleus are quantified. SEM are represented in light blue and light yellow respectively. n=number of nuclei analyzed from 3 movies of 3 embryos for each condition (See related Supplementary Movies 3 and 4). **e** Instantaneous percentage of activation after mitosis of the first active nuclei coming from active nuclei (green) and from inactive (purple). SEM are represented in light purple and light green respectively. n=number of nuclei analyzed from 3 movies of 3 embryos taken in the ventral side. **f** Mean intensity of scyl transcriptional site of the first active nuclei coming from active nuclei (green) and from inactive nuclei (purple). This quantification does not consider inactive (OFF) periods and promoter bursting. SEM are represented in light green and light purple. n=number of nuclei analyzed from 4 movies of 4 embryos. **g** One Z-plane of confocal images from immuno-smFISH with MS2 probes (red) and anti-GAF (green) on wild-type embryos and at n.c.14. Images are taken in the middle of the nuclei, where scyl_24XMS2 transcriptional sites are located. Scale bar is 5µm on the top panel and 1µm on the zoomed image.

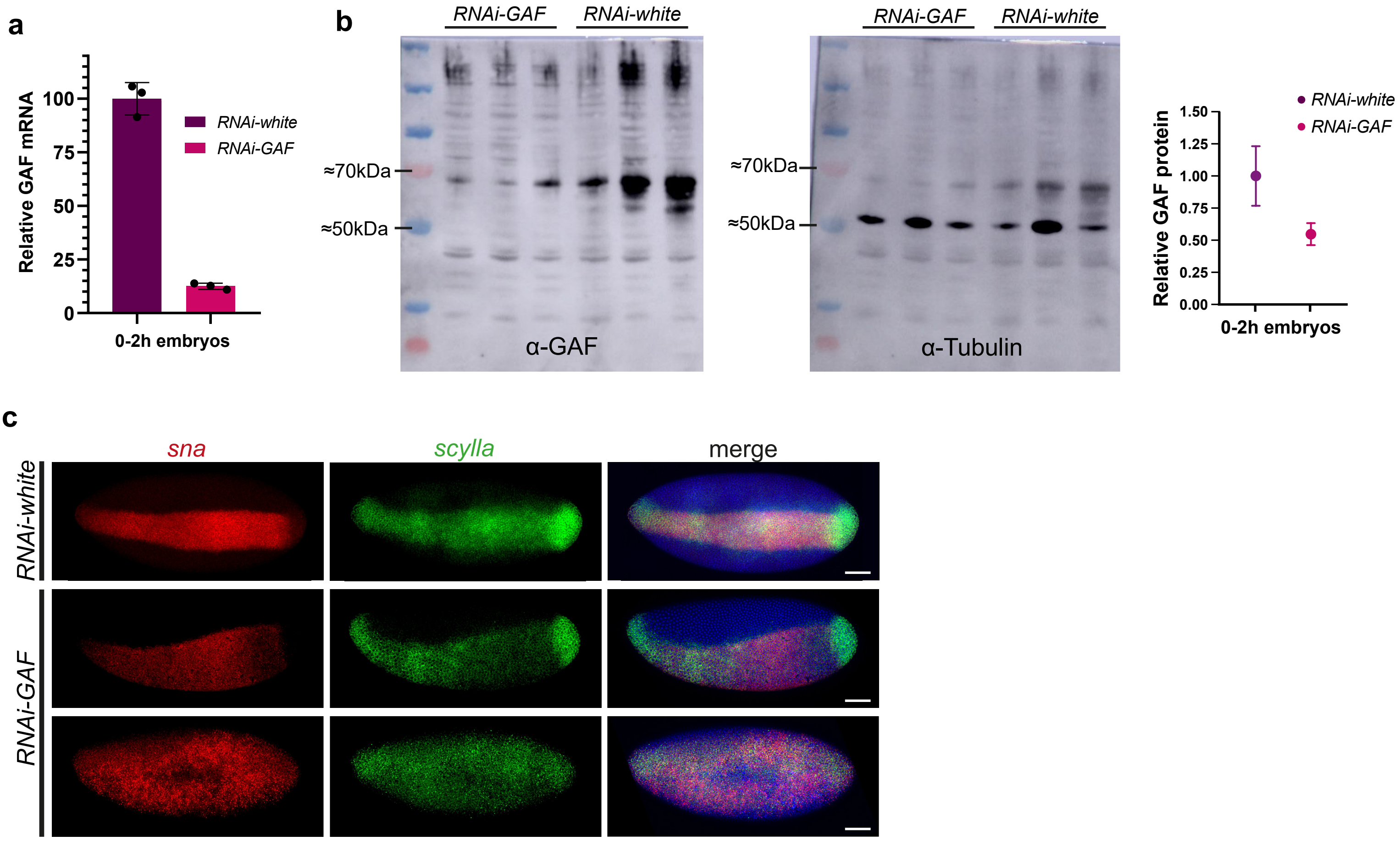

Supplementary Fig. 7. **Validation of GAF maternal reduction and consequences on scylla and snail transcription. a** (Top) Histogram of the relative amount of Ribosomal Protein L32 (RPL32) transcripts normalized GAF mRNA in RNAi-white and RNAi-GAF 0-2h embryos quantified by RT-qPCR. Technical triplicates have been performed on three independent biological replicates. Errors bars represent SD. **b** Western blot experiments of three biological replicates of RNAi-white and RNAi-GAF 0-2h embryos, with the indicated antibodies. Right panel: relative quantification of the western blot experiments. Average signal intensity from three biological replicates shown in the left panel. The signal of GAF protein was normalized on the Tubulin protein signal. Errors bars represent SD. **c** Maximum intensity projected Z-planes from confocal images from a smFISH experiment with scyl probes (red) and sna probes (green) counterstained with DAPI (blue) on RNAi-white and RNAi-GAF embryos, showing different types of phenotypes. Scale bars are 50µm.

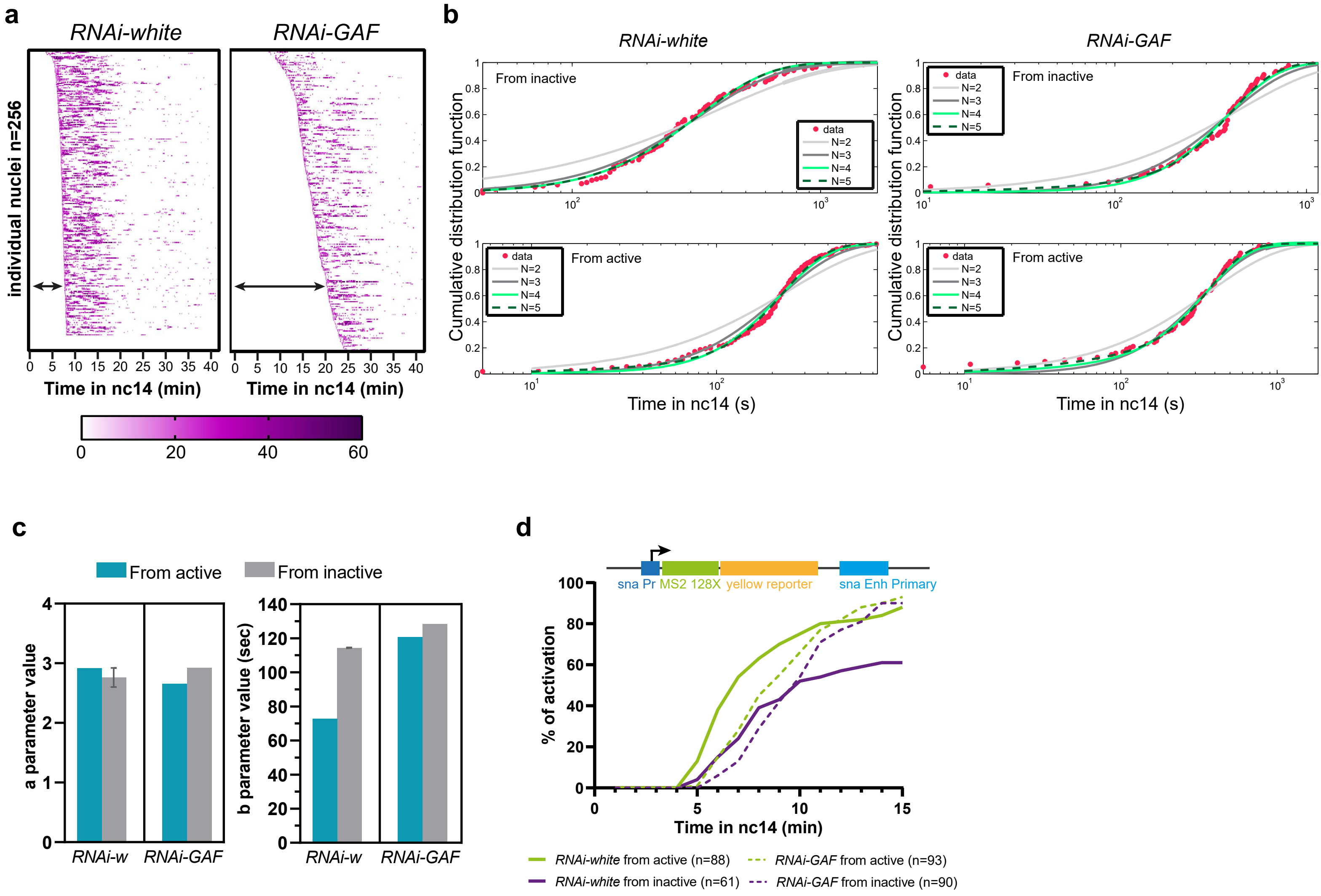

Supplementary Fig. 8. **Modeling GAF driven transcriptional memory.** **a** Heatmaps of scyl (scylla_24X-MS2_CRISPR) transcriptional site intensities of individual nuclei in RNAi-white and RNAi-GAF embryos sorted according to their first activation time after nc13 to nc14 mitosis. Black arrows represent the waiting time between mitosis and the first event of transcription of each nucleus. n: number of nuclei analyzed (a number of 256 nuclei per genotype is represented). **b** Cumulative distribution functions of multiple fits, with different number of states (N), for each genotype (RNAi-white and RNAi-GAF scylla_24X-MS2_CRISPR) and for each type of nuclei population (coming from active mothers and those coming from inactive nuclei). Note that 5 states do not significantly improve the fit, therefore we chose the number of states to be 4. The origin of time is the end of mitosis nc13 to nc14. The x axis shows the waiting times from mitosis exit to first activation (in seconds) represented in a logarithmic scale. **c** Histograms of ‘a’ and ‘b’ parameters extracted from mathematical modeling for the scyl (scylla_24X-MS2_CRISPR) gene in RNAi-white and RNAi-GAF scylla_24X-MS2_CRISPR embryos. Error intervals correspond to variation among optimal and best suboptimal fits. Parameters were extracted from 4 different movies from 4 embryos for each genotypes, representing 274 from active and 114 from inactive nuclei of RNAi-white embryos and 95 from active and 86 from inactive nuclei for RNAi-GAF embryos. **d** (Top) Schematic representing the snail-primary-enhancer_MS2 transgene. (Bottom) Cumulative activation of the first activated nuclei coming from active nuclei (green) and from inactive (purple) in RNAi-white embryos (control, solid curves) and RNAi-GAF embryos (dashed curves). n=number of pooled nuclei analyzed from 4 movies of 4 embryos.
